# Gut microbiota, angiotensin-converting enzyme, celiac disease, and risk of COVID-19 infection: a review 

**Published:** 2021

**Authors:** Fahimeh Sadat Gholam-Mostafaei, Tina Didari, Marzieh Ramandi, Reza Vafaee, Mohammad Rostami-Nejad

**Affiliations:** 1 *Gastroenterology and Liver Diseases Research Center, Research Institute for Gastroenterology and Liver Diseases, Shahid Beheshti University of Medical Sciences, Tehran, Iran*; 2 * Pharmaceutical Products Technology Development Center, Tehran University of Medical Sciences, Tehran, Iran*; 3 *Proteomics Research Center, Faculty of Paramedical Sciences, Shahid Beheshti University of Medical Sciences, Tehran, Iran*; 4 *Laser Application in Medical Sciences Research Center, Shahid Beheshti University of Medical Sciences, Tehran, Iran*; ¥ Co-first author

**Keywords:** Celiac disease, COVID-19, Gut microbiota, SARS-CoV-2, ACE2 receptor

## Abstract

Celiac disease (CD) is an autoimmune disorder of the gastrointestinal tract in a genetically susceptible person. Gluten is the most crucial trigger factor for CD, and environmental factors such as microbiota and opportunistic infection risk its pathogenesis.

Coronavirus disease 19 (COVID-19) spread rapidly and became a problem for healthcare systems worldwide. Little is known about the risk of severe COVID-19 and the role of dysbiosis among patients with CD. There is also a lack of knowledge about the effects of CD gut microbiota on COVID-19 infection. Therefore, the current review discusses the relationship between CD and risk factors such as microbiota for susceptibility to COVID-19.

## Introduction

 Celiac disease (CD) is an immune-mediated gastrointestinal (GI) disorder triggered by gluten in wheat, rye, and barley that affects genetically susceptible individuals. CD is related to small bowel disorders and associated with environmental factors in the GI tract. Pathogens such as viruses and bacteria play a critical role in small bowel mucosal immunity to increase gluten sensitivity (1). Several studies have reported that viral infection induces the production and release of tissue transglutaminase, an essential enzyme for increasing gluten immunogenicity (2, 3). Damage-associated molecular pattern (DAMP) and pathogen-associated molecular pattern (PAMP) can play a crucial role in T-cell activation in the inflammatory pathway to the pathogenesis of CD (4). CD is common among both genders at any age. The genetic background of familial CD is more than 10% in people with HLA-DQ2 or HLA-DQ8 (5, 6).

The World Health Organization (WHO) has confirmed that the COVID-19 pandemic (or coronavirus pandemic) in March 2020 was caused by SARS-COV-2 (Severe acute respiratory syndrome coronavirus-corona virus-2) (7). The first serious discussion reported novel coronavirus (SARS-COV-2) in December 2019 in Wuhan, China. SARS-COV-2 is associated with an increased risk of acute respiratory distress syndrome (ARDS) by airborne transmission (8). The increasing mortality rate of the ongoing COVID-19 pandemic is a growing healthcare concern worldwide. The most at-risk groups for ARDS of COVID-19 are geriatrics and patients with an underlying health condition such as obesity, cardiovascular disease, pulmonary dysfunction, hypertension, chronic kidney disease, immunocompromised cases, and Type 1 diabetes mellitus (9). CD has an overlap in genetic and environmental risk factors with type 1 diabetes, but knowledge about the risk of COVID-19 for celiac patients is lacking. This study provides new insight into the relationship between COVID-19 and CD. 

## Methods

**Search strategy**

Electronic databases such as Pubmed, Web of Science, Scopus, EMBASE, Cochrane Library, and Google Scholar were searched for articles published from November 1996 to November 2020. The applied MESH terms were “Celiac’s Disease”, “Gluten Enteropathy”, “Gluten-Sensitive Enteropathy”, “Nontropical Sprue”, “Celiac Sprue”, “CD”, “2019 novel coronavirus disease”, “COVID19”, “COVID-19 pandemic”, “SARS-CoV-2 infection”, “COVID-19 virus disease”, “2019 novel coronavirus infection”, “2019-nCoV infection”, “coronavirus disease 2019”, “coronavirus disease-19”, “2019-nCoV disease”, and “COVID-19 virus infection”. Moreover, EMTREE terms used in the present review were; “adult celiac disease”, “adult coeliac disease”, “celiac sprue”, “celiac syndrome”, “coeliac disease”, “coeliac sprue”, “coeliac syndrome”, “coeliaky”, “gluten induced enteropathy”, “gluten intolerance”, “2019-nCoV disease”, “2019-nCoV infection”, “COVID 19”, “nCoV 2019 disease”, “nCoV 2019 infection”, “novel coronavirus 2019 disease”, “novel coronavirus 2019 infection”, “novel coronavirus disease 2019”, “novel coronavirus infection 2019”, “Wuhan coronavirus disease” and “Wuhan coronavirus infection”. Two researchers reviewed the reference lists of all included papers to find additional studies.

## Results

**Novel coronavirus disease 19 (COVID-19)**

Coronaviruses are categorized as RNA viruses that cause pulmonary infection from mild to severe in both mammals and birds. They form the subfamily of Orthocoronavirinae in the Coronaviridae family. The coronavirus responsible for lethal atypical pneumonia in the 2019 pandemic is coronavirus disease-19 (Covid-19). The novel coronavirus is defined as the Severe Acute Respiratory Syndrome of Corona Virus 2 (SARS-CoV-2). This viral respiratory disease is a family member of Severe Acute Respiratory Syndrome (SARS) and Middle East Respiratory Syndrome (MERS) of previous pandemics in 2003 and 2012, respectively (10, 11). As an airborne virus, SARS-CoV-2 was transmitted quickly from China and caused a pandemic, namely COVID-19, that has affected economies and public health systems worldwide. Clinical symptoms of COVID-19 patients range from mild to severe levels. The coronaviruses cause zoonotic infections ranging from the low grade of the common cold to pulmonary dysfunction in critically ill individuals (12). Many persons are asymptomatic carriers, but some cases are at risk of acute respiratory distress syndrome (13). The common symptoms of COVID-19 are usually fever, cough, sore throat, breathlessness, fatigue, malaise, loss of smell or taste (14), pulmonary infection, and gastrointestinal (GI) symptoms such as abdominal pain, diarrhea, nausea, vomiting, and impaired liver function (15). 

**Association between coronavirus and angiotensin-converting enzyme (ACE)**

Coronaviruses are composed of spike proteins (S), lipid bilayer cover, envelope (E), and membrane proteins (M) plus single-strand RNA genome (positive-sense). They have one of the largest genome sizes among RNA viruses (26-32 kb). The club shape of spikes has an essential role in sticking their receptors in the body (13, 16, 17). In combination with M and E, S proteins provide effective binding sites to interact with host cell receptors. Angiotensin-converting enzyme (ACE) and angiotensin-converting enzyme 2 (ACE2) as peptidase are crucial to regulating cellular interactions in the cardiovascular system. The degradation of precursors by various peptidase generates ACE, which plays a vital role in the renin-angiotensin-aldosterone system (RAAS) to regulate blood pressure by controlling fluid volume and cutting pro-peptide angiotensin-1 (Ang I) into active peptide angiotensin-2 (Ang II) (18). ACE requires zinc ions to catalyze peptide hydrolysis and is potentially a vasodilator to degrade bradykinin in the kinin-kallikrein system and chloride anions to enzyme structure stabilization (19). The homologous form of ACE is ACE2 for biological responses such as microvascular capillary, inflammatory pathways, and wound healing. ACE2 also has a protective effect against tissue injury by attenuating the harmful effects of Ang II (20). ACE2 expression is mainly found on the surface of endothelial cells of the urinary system, cardiopulmonary organs, male reproductive systems, gastrointestinal tract, and liver (21). One of the most important receptors of the host involved in binding spike proteins of SARS-CoV-2 to target cells is ACE2 (22). The S glycoprotein of SARS-CoV-2 contains two subunits; the S1 subunit is the receptor-binding domain (RBD), and the S2 subunit is for viral fusion to the host cells. Investigators have claimed that the RBD of the S1 and S2 subunits of SARS-CoV-2 bind to ACE2 with high affinity and infected cells with a higher content of ACE2, such as adipocytes, endothelial cells, enterocytes, and pneumocyte cells type II. Cardiomyocytes then blockade the normal functions of cells and cause multi-organ dysfunctions (MODS). ACE2 is considered a doorway for SARS-CoV-2 to enter the host cells. Moreover, ACE2 is higher in cardiovascular disease, hypertension, and diabetes cases than in healthy persons (23). It also plays a critical role in the inflammation and microbial composition of the gastrointestinal tract. A study on gnotobiotic rats showed 

**Figure 1:**
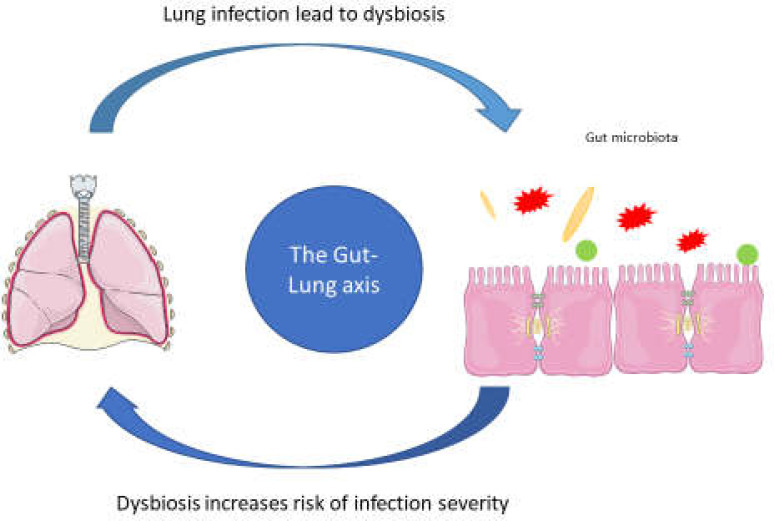
Gut microbiota imbalance is associated with an increased risk of infection severity

that changes in the gut microbial composition could be a factor modulating colonic Ace2 expression and influencing the COVID-19 infection through the gut-lung axis.

**The effect of SARS-CoV-2 on gut microbiota**

Gut microbiota in the human population are influenced by genes, environment, and nutrition. Dysbiosis is defined as an alteration in the microbiome structure that changes the composition of the host microorganism community. It affects pathogens exposure, diet changes, antibiotic administration, and genetically susceptible hosts (24, 25). Gut dysbiosis is defined as gut microbiome alterations leading to gastrointestinal discomfort. It is associated with some diseases such as inflammatory bowel disease (IBD), type 2 diabetes, cardiovascular disease, and CD (26-29). Researchers have shown that dysbiosis could be the reason for severe illness from COVID-19 (Figure1). A decrease in gut microbiota diversity in older adults may possibly lead to severe COVID-19 infection and an increased mortality rate (30). 

It seems that about 50% of SARS-CoV-2 positive cases reported gastrointestinal symptoms (31). Interestingly, it has been proven that cross-talk between gastrointestinal impairment is related to the viral load of SARS-CoV-2 in the lung and referred to as the “gut-lung axis”. It is suggested that lung inflammation and cytokine storm during COVID-19 affect the human microbiome (32). 

After pulmonary alleviation in discharged patients with COVID-19, stool specimens remained viral positive. Overexpression of ACE2 makes it a candidate to bind, fuse, and enter SARS-CoV-2 to the host cells in the membrane of intestinal colonocytes and enterocytes (33, 34). In an observational study of a hospitalized patient with COVID-19, Zhou et al. showed that cases with positive metagenomic sequencing of SARS-CoV-2 indicated higher levels of *Morganella morganii, Streptococcus infantis, Collinsella aerofaciens, *and* Collinsella tanakaei*. The metabolism of amino acids (such as L-serine), nucleotides (synthesis of adenosine and guanosine), and glycolysis were significantly increased in this group. In contrast, healthy subjects or patients with a lower viral load of SARS-CoV-2 showed an overpopulation of short-chain fatty acid synthesis bacteria, such as *Alistipes onderdonkii, Bacteroides stercoris, Parabacteroides merdae, and Lachnospiraceae bacterium 1_1_57FAA* in fecal samples. Zhou et al. concluded that opportunistic bacteria was significantly increased with higher infection of SARS-CoV-2 in the patient arm of the study than the other arm which had no or lower content of SARS-CoV-2 disease. Thus, gut inflammation in SARS-CoV-2 patients showed a combination of factors that guaranteed the opportunistic bacteria survival rate. These markers include L-serine biosynthesis, cytokine induction of 8-hydroxydeoxy guanosine (8-OHdG), oxidative stress marker of DNA, and overexpression of adenosis (35). 

**Celiac disease and gut microbiota**

Celiac disease is known as gluten-sensitive enteropathy or celiac sprue. It is an immune reaction to gluten consumption, inflames the small intestine, and causes malabsorption of nutrients. It is an autoimmune disease with a multifactorial condition. Celiac disease is more common among patients with diabetes, colitis, Addison’s disease, thyroid dysfunction, down or Turner syndrome, or a genetic background of celiac disease (36). A gluten-free diet is the only treatment for celiac patients, because they have a hypersensitivity to gliadin and glutenin proteins in gluten. Factors involved in celiac disease include genetics, prenatal factors, infections, and microbiota (37). In patients genetically susceptible to celiac, HLA-DQ haplotypes are one of the leading indicators of developing CD that affects gut microbiota (38). Early-onset of CD is found among infants under two years of age. Type of feeding, childbirth mode, and antibiotic administration in mothers during pregnancy increase the risk of CD. The risk of genetic CD is reduced in breastfed infants by the overpopulation of *Bifidobacterium* and *Streptococcus* species as anti-inflammatory bacteria. Proteobacteria overexpression has also been found in this group (39). Also, *Bacteroides* species such as *B.vultagus, B.Prevotella, E.coli, C.lituseburense, C.histoliticum*, *Firmicutes*, *Proteobacteria*, *Actinobacteria*, *Enterococcus*, *Propionibacterium, and Subdoligranulum* species were increased in the microbiota of CD infants (40-44). Many studies have revealed that exposure to microbial infection changes the permeability of the intestine and reduces autoimmunity and tolerability of enterocytes. Harnett et al. showed an overpopulation of the opportunistic bacteria *Morganella morganii* in the gut microbiome of CD cases (45). Moreover, sequencing of the 16S rRNA gene in children and adults suffering from CD revealed that *Prevotella* spp. and *Streptococcus* spp. were significantly higher in CD subjects compared to CD cases receiving probiotic supplements (46). In addition, the abundant genus of *Streptococcus* and *Collinsella* were found as specific bacteria for CD pathogenesis (29). 

**Risk of opportunistic infection in celiac disease**

CD is considered an autoimmune dysregulated condition and activates both adaptive and innate immune systems (47). Many studies have reported that celiac disease is associated with opportunistic infections such as *C. difficile*, sepsis, and *streptococcal *infection, and increases the risk of hospitalization for influenza (48-50). However, there is not enough evidence of the novel coronavirus risk in celiac disease. Investigating the risk of COVID-19 infection among CD patients is complex, because the role of the GFD and change in gut microbiota and their effects on the immune system are not well identified.

**Prevalence of SARS-CoV-2 and Celiac disease**

Few studies have investigated the risk of COVID-19 in celiac patients compared to healthy cases. Zhen et al. found that CD patients had similar odds of COVID-19 infection compared with the general population (51). Lionetti et al. examined the prevalence of COVID-19 infection in patients aged 0–16 years and found no significant increase in the prevalence of COVID-19 compared with the controls (52). The Celiac Center of Columbia University has decided to launch a new registry of Surveillance Epidemiology of Coronavirus Under Research Exclusion in Celiac patients (SECURE-Celiac project). This study is based on the long-term gluten-free diet that increases the risk of influenza and bacterial pneumonia in celiac cases during the COVID-19 pandemic, concerns about the gluten-free diet, and the elevated risk of COVID-19 among CD patients (53). The cohort study of twenty-one COVID-19 patients who suffered from CD or refractory celiac disease (RCD) demonstrated a lower risk of COVID-19 infection in both groups. It suggested that the presence of HLA-DQ2 in 90% of CD subjects reduced T-helper interactions, down-regulated cytokine production such as IL-4 and IFN γ, and may alleviate COVID-19 symptoms among CD or RCD cases (54). Until now, the exact role of the cellular pathway and severity of COVID-19 in CD cases is unclear, and further research is needed. 

**Intestinal dysbiosis as a risk factor for COVID-19 in celiac patients**

Intestinal epithelium cells are barriers to protect the mucosa from invading microorganisms. Investigators have claimed that commensal bacteria are essential to modulating the immune system, improving antiviral immune function, elevating mucosal immune responses such as T cell activation, producing IgA, regulating homeostasis, and generating antimicrobial peptides by the intestinal epithelial cell (55). Changes in microbial composition activate the innate immune system and pro-inflammatory cytokines, leading to epithelial barrier disruption and an increased transfer of gluten immunogenic peptides and activation of adaptive Th1/Th17 pathways; this causes villous atrophy in CD patients. Accordingly, disruption in the intestinal epithelium barrier integrity due to dysbiosis may cause the translocation of COVID-19 viruses from the pulmonary system into the intestinal lumen by the circulatory and lymphatic systems (30).

The gut-lung axis is the cross-talk between the pulmonary system and gut microbiota to maintain host cells' immune function and cellular homeostasis. The interaction between intestinal microbiota and lung inflammation may affect COVID-19 severity in patients with underlying diseases. A search of the literature revealed that lung inflammation during SARS-CoV-2 infection leads to alterations in gut microbiota. Thus, COVID-19 may influence inflammatory cytokines production and cytokine storm induction in the gut. Aging, underlying health condition, reduction of the immune response, and dysbiosis are risk factors for COVID-19 severity. It is suggested that translocation of gut barrier integrity is interrupted during dysbiosis and leads to SARS-CoV-2 translocation from alveolar cells into the intestinal tract via the bloodstream (30, 56, 57). Many studies have reported that dysbiosis might occur in the duodenal and stool of patients with celiac (29, 58, 59). Furthermore, dysbiosis in COVID-19 may play a critical role in severe clinical symptoms such as pneumonia and ARDS in immune-compromised patients. Elevated ACE2 levels were found in patients with pro-inflammatory gut microbiota such as IBD cases. It created most favorable conditions for SARS-CoV-2 infection, but no study has considered the ACE2 content of CD patients suffering from COVID-19 (60-62).

## Conclusion

Research has shown that SARS-CoV-2 infection triggers ACE2 in the GI tract and disrupts epithelial cells to invade enterocytes and change gut microbiota (34). The gut-lung axis as an interaction between microbiota and the overexpression of cytokines may lead to GI dysfunction during COVID-19 (63). Although some studies have reported that the prevalence of COVID-19 infection among CD patients on GFD is similar to that among healthy subjects, there is a lack of knowledge about the risk of this infection at the time of diagnosis. The underlying mechanism of gut microbiota alteration, ACE2 expression level, dysbiosis, and gut-lung axis among CD cases with COVID-19 infection is still unclear, and there is no published clinical data on the regulation of gut microbiota in CD patients as a treatment of COVID-19. Furthermore, improvement of gut microbiota has been demonstrated as a treatment option for CD cases (64). Despite SARS-CoV2 RNA identification in fecal samples from COVID-19 subjects, active SARS-CoV-2 infection of carriers were not found (65). Thus, further research is needed to evaluate CD cases of gastrointestinal change who suffered from COVID-19.

## Conflict of interests

The authors declare that they have no conflict of interest.
